# Functional compatibility between Purkinje cell axon branches and their target neurons in the cerebellum

**DOI:** 10.18632/oncotarget.19770

**Published:** 2017-08-01

**Authors:** Zhilai Yang, Na Chen, Rongjing Ge, Hao Qian, Jin-Hui Wang

**Affiliations:** ^1^ Institute of Biophysics, Chinese Academy of Sciences, Beijing 100101, China; ^2^ College of Life Science, University of Chinese Academy of Sciences, Beijing 100049, China; ^3^ Qingdao University, School of Pharmacy, Shandong 266021, China; ^4^ Department of Physiology, Bengbu Medical College, Bengbu 233000, China; ^5^ Department of Anesthesiology, The First Affiliated Hospital of Anhui Medical University, Hefei 230022, China

**Keywords:** axon, neuron, action potential, synaptic transmission, Purkinje cell

## Abstract

A neuron sprouts an axon, and its branches to innervate many target neurons that are divergent in their functions. In order to efficiently regulate the diversified cells, the axon branches should differentiate functionally to be compatible with their target neurons, i.e., a function compatibility between presynaptic and postsynaptic partners. We have examined this hypothesis by using electrophysiological method in the cerebellum, in which the main axon of Purkinje cell projected to deep nucleus cells and the recurrent axons innervated the adjacent Purkinje cells. The fidelity of spike propagation is superior in the recurrent branches than the main axon. The capabilities of encoding spikes and processing GABAergic inputs are advanced in Purkinje cells versus deep nucleus cells. The functional differences among Purkinje's axonal branches and their postsynaptic neurons are preset by the variable dynamics of their voltage-gated sodium channels. In addition, activity strengths between presynaptic and postsynaptic partners are proportionally correlated, i.e., active axonal branches innervate active target neurons, or vice versa. The physiological impact of the functional compatibility is to make the neurons in their circuits to be activated appropriately. In conclusion, each cerebellar Purkinje cell sprouts the differentiated axon branches to be compatible with the diversified target cells in their functions, in order to construct the homeostatic and efficient units for their coordinated activity in neural circuits.

## INTRODUCTION

In terms of functional interaction between the neurons, the efficient and coordinated relationships between presynaptic axons and postsynaptic neurons are compatible in their activity strengths, i.e., active axons innervate active neurons, or vice versa [1–3]. Otherwise, active axons drive inactive neurons leading to ineffective energy-cost, and inactive axons cannot activate active target neurons forming the silent partner. Moreover, each neuron sprouts an axon, and its axonal branches innervate numerous neurons. The sequential spikes generated on each neuron propagate through its axonal branches to the terminals and in turn regulate their diversified postsynaptic neurons [4–8]. The activity diversity of postsynaptic cells may require the functional state of presynaptic axonal branches to be differentiated, in order to form compatible relationship between presynaptic axonal branches and postsynaptic neurons in activity strengths, i.e., a functional compatibility between presynaptic and postsynaptic partners [2]. In other words, each neuron uses its axonal branches as the fractional diverters and regulates its postsynaptic cells appropriately. We have examined this hypothesis at the units that consisted of a Purkinje cell and its target neurons in the mouse cerebellum.

Each cerebellar Purkinje neuron sprouts a main axon and a few recurrent axonal branches that are GABAergic. The main axon innervates the neurons in the deep nucleus and the recurrent axons project to adjacent Purkinje cells [9–23]. In cerebellar slices, we have investigated whether the functional status was differential among Purkinje cell axon branches and among their target cells, as well as whether presynaptic and postsynaptic partners were functionally compatible. The functional states of presynaptic axonal branches were evaluated based on their abilities to propagate spikes and to release transmitters. The functions of the target cells innervated by these axonal branches were evaluated based on their capabilities to produce spikes and to respond to transmitter. In addition to analyzing their functional differentiation, we plotted a correlation of activity levels in each of presynaptic and postsynaptic partners. A proportional positive correlation implies the function compatibility between presynaptic and postsynaptic partners.

## RESULTS

### Spike propagation is superior in recurrent axons than main axons of cerebellar Purkinje cells

**Figure 1:**
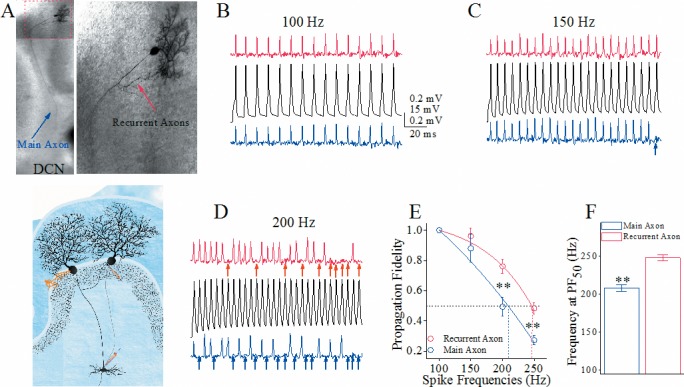
The spike propagation fidelity on the main axons and recurrent branches of cerebellar Purkinje cells (PC) is distinct **(A)** Top-left panel shows a neurobiotin-labeled PC whose main axon extends to deep cerebellar nucleus (DCN) and recurrent axons to adjacent PCs. Top-right is an enlarged photo. Bottom shows whole-cell recording on PC as well as two loose-patch recordings on its main axon and recurrent axon. **(B-D)** Black traces illustrate somatic spikes induced by whole-cell recording pipette at PC (middle) and spikes recorded by loose-patch on recurrent axons (red traces in top) and on main axons (blues in bottom). Somatic spikes are induced by sequential depolarization pulses from 100 Hz to 250 Hz. The arrows under loose-patch recorded signals show the failure of spike propagation on the axons. Calibration bars are 15 mV (for whole-cell spikes)/0.2 mV (loose-patch spikes) and 20 ms. **(E)** shows somatic spike frequency versus propagation fidelity on recurrent axons (red symbols) and main axons (blues; asterisks, p<0.01, n=7), i.e., the ratio of axonal spikes to somatic ones. Spike frequency at 50% propagation fidelity (PF_50_ showed as dash line) is defined as spike-propagation efficiency. **(F)** shows spike frequencies at PF_50_ on recurrent axon (red bar) and main axon (blue; asterisks, p<0.01).

One of major functions for the axons is propagating sequential spikes [24]. Spike propagation fidelity was assessed by a ratio of the spikes propagated on axons to the spikes evoked at somata [25–27]. To monitor the spike propagation on the axons of each Purkinje cell, we evoked the spikes on its soma by depolarization pulses through a whole-cell pipette, and recorded the spikes propagated to the remote ends of its main axon and recurrent axon by two loose-patch pipettes simultaneously (Figure 1A). By comparing the spikes on soma (middle black traces in Figure 1B–D) and axonal branches (top red traces for recurrent axon and bottom blue ones for main axon), we can see that the spike propagation fails more on main axons than recurrent axons when the spikes are above 150 Hz. The spike propagation fidelity versus spike frequency in Figure 1E illustrates that the frequency-dependent fidelity of spike propagation is higher in the recurrent axonal branches (red symbols) than the main axons (blue ones; p<0.01, n=10 pairs). Spike frequencies at 50% of propagation fidelity (PF_50_) that merit spike propagation fidelity are 248±3.9 Hz on the recurrent axons and 208±4.4 Hz on the main axon (Figure 1F, p<0.01). The superior spike propagation on the recurrent axons versus the main axons indicates a functional differentiation of axonal branches from Purkinje cells.

**Figure 2:**
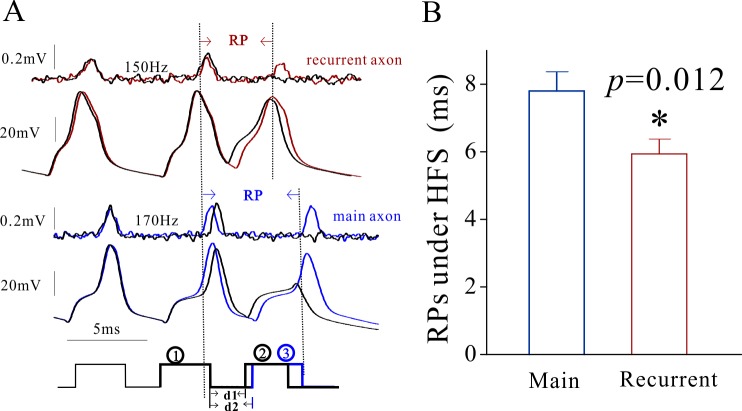
The refractory periods of voltage-gated sodium channels on the main axon and recurrent axons of cerebellar Purkinje cells (PC) is distinct The refractory periods (RP) were recorded on the remote ends of these axonal branches by loose-patches while the spikes were induced at the PC somata by whole-cell recording. **(A)** illustrates the waveforms of RP measurements from the recurrent axon (red/black traces in top panels) and the main axon (blue/black traces in bottom panels). **(B)** shows the comparison of RP values from main axons (blue bar; n=11) and recurrent axons (red bar; n=11, p=0.012). It is noteworthy that Figure 2A presents a single trace in order to have a clear demonstration of spikes.

The mechanism underlying the differences of spike propagation fidelity between axonal branches is likely based on the distinct dynamics of voltage-gated sodium channels (VGSC) on the two subcellular compartments since VGSCs play an essential role in spike propagation [27]. VGSCs’ dynamics was assessed by measuring their refractory periods (RP) at the remote ends of recurrent and main axons with loose-patches (Methods). As spike propagation failure occurs in the late stage of spiking (Figure 1), we measured RPs after sequential spikes (Figure 2). RP values (Figure 2A) appear longer in main axons (blue trace) than recurrent axons (red). The statistical analysis indicates that RP values are 7.8±0.3 ms for main axons (blue bar in Figure 2B) and 5.92±0.25 ms for recurrent axons (red, p=0.012, n=11). These relatively quick recoveries of VGSC inactivation and subsequent reactivation on recurrent axons lead to their greater spike propagation, compared with those on main axons.

**Figure 3:**
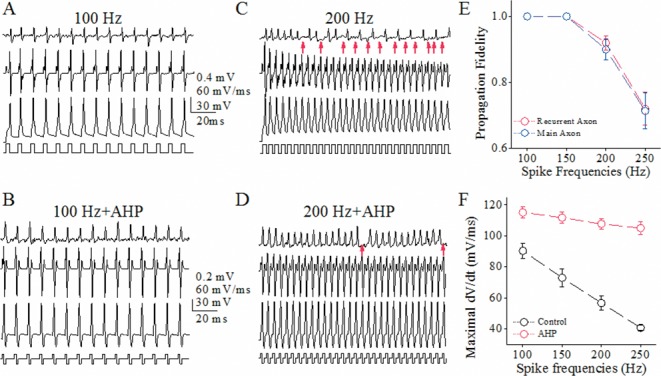
After-hyperpolarization raises spike propagation fidelity on main axons up to that on recurrent branches of cerebellar Purkinje cells (PC) **(A-B)** show sequential spikes induced by depolarization pulses (100 Hz in A) and the mixed pulses of depolarization and hyperpolarization (100 Hz in B). The traces from top to bottom are spikelets on main axon, dV/dt values, spikes on PC soma and pulse patterns. **(C-D)** show sequential spikes induced by depolarization pulses (200 Hz in C) and the mixes of depolarization and hyperpolarization (200 Hz in D). The traces from top to bottom are spikelets on main axon, dV/dt values, spikes on PC soma and pulse patterns. Red arrows indicate the failures of spike propagation. **(E)** shows somatic spike frequency versus propagation fidelity on the recurrent axons (red symbols) and main axons (blues; n=10), i.e., a ratio of axonal spikes to somatic ones. **(F)** shows maximal dV/dt values versus spike frequencies on recurrent axons (red bar) and main axons (blue; asterisks, p<0.01; n=10).

This indication was studied by seeing an effect of rescuing VGSC function on spike propagation. As after-hyperpolarization (AHP) lowered spike threshold potentials and refractory periods [27, 28], we investigated the effect of AHP on spike propagation (Figure 3). AHP appears to raise spike propagation fidelity at its high frequency on main axons (Figure 3A–D). Figure 3E shows that AHP upregulates spike propagation fidelity on main axons closely to that on recurrent axons. This increase of spike propagation fidelity is due to strengthening VGSC reactivation, since AHP elevates the dV/dt values of spike rising slope (Figure 3F), an index of synchronous activation of VGSCs [27]. This result also indicates that the lengths of main axon versus recurrent axon do not affect the difference in their fidelity of propagating spikes.

In summary, axon branches sprouted from cerebellar Purkinje cells are functionally differentiated, and the activity is superior in recurrent axons than main axons, which is based on the different dynamics of their VGSCs. We subsequently studied whether this functional differentiation was present in the target neurons of these axonal branches.

### Spiking capability is superior in Purkinje cells than deep nucleus cells in the cerebellum

**Figure 4:**
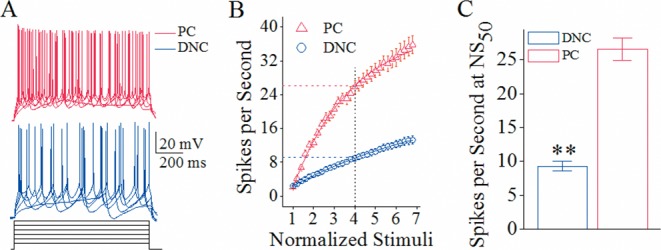
The spiking ability is higher at cerebellar Purkinje cells (PC) than deep nucleus cells (DNC) **(A)** Superimposed waveforms show sequential spikes induced by depolarization pulses in various intensities at a Purkinje cell (red traces) and deep nucleus cell (blue traces). Calibration bars are 20 mV and 200 ms. **(B)** illustrates spike per second versus normalized stimuli, i.e., input-output curves, for Purkinje cells (red symbols, n=10) and deep nucleus cells (blues, n=10). Spikes per second at 50% of normalized stimuli (NS_50_) reflect the ability of encoding spikes. The normalized stimuli are based on the threshold intensity of evoke a spike during 200 ms, and the step of intensity increase is 10% of threshold intensity. **(C)** shows spike per second at NS_50_ from Purkinje cells (red bar) and deep nucleus cells (blue; asterisks, p<0.01).

In Purkinje cells and deep nucleus cells that are the target cells of recurrent axons and main axons, respectively, we assessed their spiking ability by measuring relationship between stimulus intensities and spikes (input-output curve; [29]. Depolarization pulses (black traces in Figure 4A) appear to induce more spikes at Purkinje cells (reds) than deep nucleus cells (blues). Figure 4B shows spikes per second versus normalized stimuli for Purkinje cells (red symbols, n=10) and deep nucleus cells (blues, n=10). Spikes per second at 50% normalized stimuli (NS_50_) that merit spiking capability are 26.53±1.65 on Purkinje cells and 9.3±0.71 on deep nucleus cells (p<0.01 in Figure 4C). The cerebellar Purkinje cells possess higher ability to encode spikes, compared to deep nucleus cells. Therefore, Purkinje cell's target cells are functionally differentiated.

**Figure 5:**
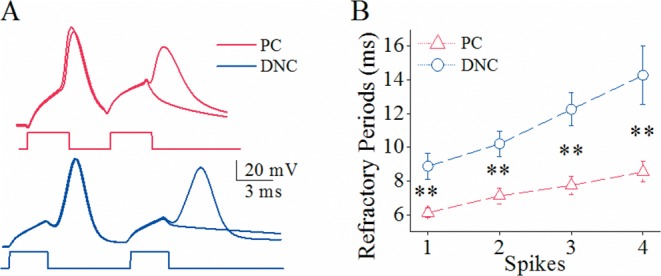
The spike refractory periods is shorter at cerebellar Purkinje cells (PC) than deep nucleus cells (DNC) **(A)** The superimposed waveforms illustrate longer refractory periods (RP) at a deep nucleus cell (blue traces) than a Purkinje cell (reds). **(B)** shows statistical data for RP values of spikes 1-4 at PCs (red symbols, n=10) and DNCs (blues, n=10; asterisks, p<0.01).

The input-output curve in spike production is presumably controlled by VGSC dynamics [30–32]. The differences of spiking ability on these two groups of the neurons may be due to differential VGSC dynamics. As spike refractory period (RP) is an index of VGSC dynamics [33–35], we measured RP at cerebellar Purkinje cells and deep nucleus cells (Figure 5). RP appears longer at deep nucleus cells than Purkinje cells (Figure 5A). RP values for spikes 1-4 are 6.1±0.1, 6.9±0.13, 7.5±0.21 and 7.9±0.25 ms at Purkinje cells, and are 8.8±0.4, 10.5±0.39, 12.2±0.5 and 13.5±0.9 ms at deep nucleus cells. RP values for corresponding spikes in these two kinds of the neurons are statistically different (two asterisks, p<0.01; n=10).

In outline, the target cells of cerebellar Purkinje cells are functionally differentiated, and Purkinje cells are superior to deep nucleus cells, which is based on their different VGSC dynamics. In addition, we analyzed transmitter release to assess presynaptic axon function as well as receptor responsiveness to estimate postsynaptic neuronal function. The following experiments show whether presynaptic transmitter release and postsynaptic receptor function in main axons to deep nucleus cells versus recurrent axons to Purkinje cells are functionally differentiated.

### GABA release and receptor responsiveness are superior in recurrent axons to Purkinje cells

In the presynaptic and postsynaptic partners of main axons to deep nucleus cells versus recurrent axons to Purkinje cells, GABA release was assessed by analyzing the frequency of spontaneous inhibitory postsynaptic currents (sIPSC) and their receptor responsiveness to GABA was evaluated by the amplitude of sIPSCs [36, 37]. sIPSCs were recorded on cerebellar Purkinje cells and deep nucleus cells.

**Figure 6:**
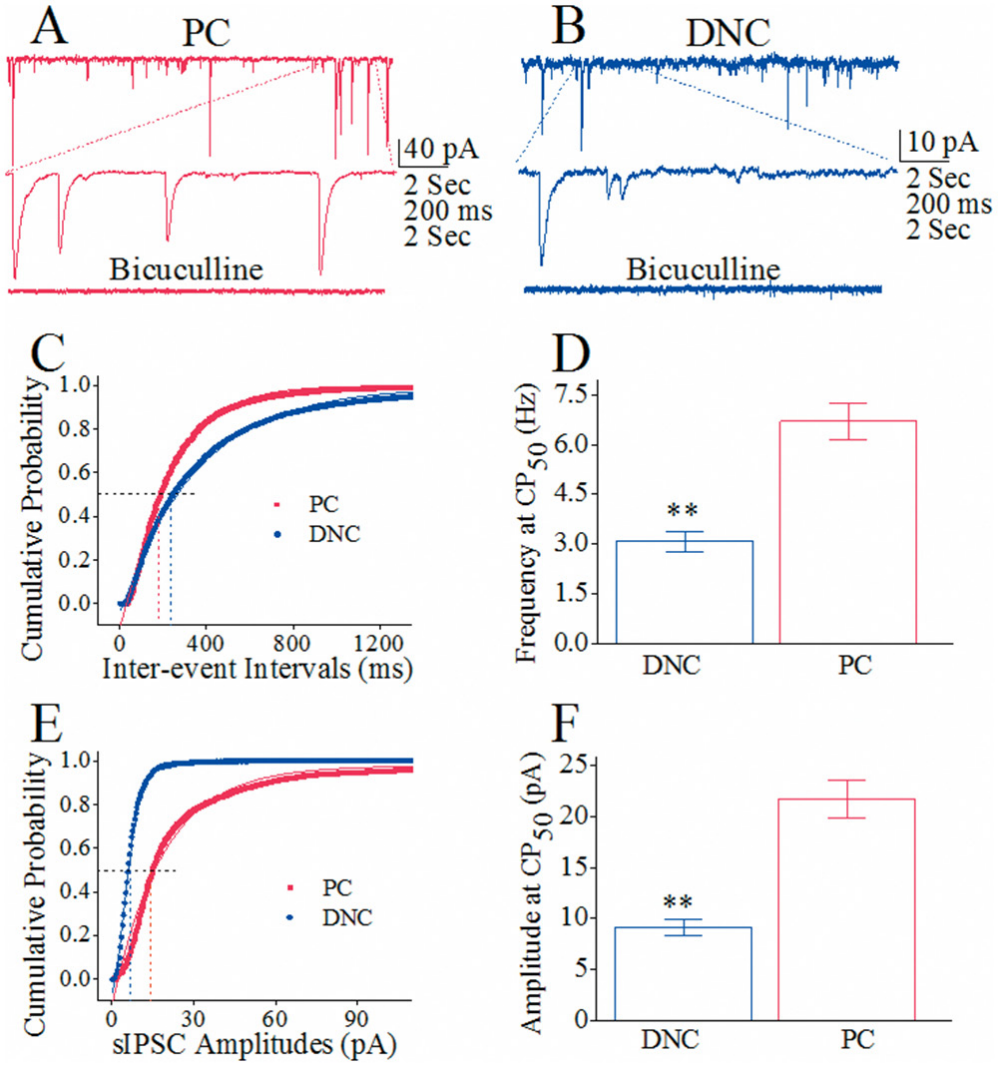
The comparison in the activity of GABAergic synapse on Purkinje cells (PC) and deep nucleus cells (DNC) The activities of GABAergic synapses are evaluated by recording sIPSCs on these cells under voltage-clamp in the presence of CNQX and D-AP5. **(A)** sIPSCs recorded from a PC in the control and presence of 10 μM Bicuculline (bottom trace). **(B)** sIPSCs recorded from a DNC in the control and presence of Bicuculline (bottom trace). **(C)** Cumulative probability vs. inter-event intervals for DNCs (blue symbols, n=10) and PCs (red ones, n=10), in which sIPSC frequencies (1/inter-event intervals) at 50% of cumulative probability (CP_50_) reflect presynaptic GABA release. **(D)** shows sIPSC frequencies at CP_50_ from recurrent axon (red bar) and main axon (blue bar; asterisks, p<0.01). **(E)** illustrates cumulative probability versus sIPSC amplitudes for DNCs (blue symbols, n=10) and PCs (reds, n=10), where sIPSC amplitudes at 50% of cumulative probability (CP_50_, dash line) present postsynaptic responsiveness. **(F)** shows sIPSC amplitudes at CP_50_ on Purkinje cells (red bar) and deep nucleus cells (blue bar; asterisks, p<0.01).

sIPSC amplitudes and frequencies appear to be higher on Purkinje cells than deep nucleus cells (Figure 6A–B). Figure 6C shows cumulative probability versus inter-event intervals in Purkinje cells (red symbols; n=10) and deep nucleus cells (blues; n=10). sIPSC frequencies at 50% cumulative probability (CP_50_) that merit GABA release are 6.78±0.62 Hz from recurrent axons and 3.1±0.3 Hz from main axons (p<0.01; Figure 6D). The superior GABA release from recurrent axons than main axons further implies a functional differentiation of axonal branches from Purkinje cells. Figure 6E shows cumulative probability versus sIPSC amplitudes in Purkinje cells (red symbols) than deep nucleus cells (blue). sIPSC amplitudes at 50% cumulative probability (CP_50_) that merit GABA receptor responses are 24.1±2.1 pA on Purkinje cells and 9.33±0.91 pA on deep nucleus cells (p<0.01 in Figure 6F). The superior responses of Purkinje cells to GABA than of deep nucleus cells further indicates a functional differentiation of Purkinje's target cells.

The abilities of spike encoding and synaptic transmission in presynaptic and postsynaptic entities are differentiated. Compared the functional states of axonal branches and their target cells in the units of a Purkinje cell to postsynaptic neurons (Figures 1, 2, 3, 4, 5, 6), we found that the activity strengths were superior in a pathway from recurrent axons to Purkinje cells than a pathway from main axon to deep nucleus cells. We subsequently examined whether the activity strengths between presynaptic and postsynaptic partners were compatible by analyzing their functional correlations.

### The functional states between axonal branches and their target neurons are linearly correlated

**Figure 7:**
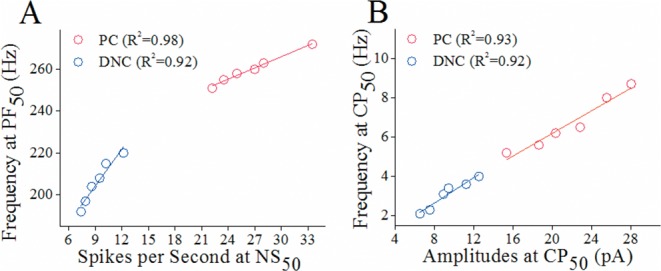
The correlations of functional status between presynaptic axonal branches of Purkinje cells (PC) and their target cells **(A)** shows the linear correlations between presynaptic spike frequencies at PF_50_ and postsynaptic spikes per second at NS_50_ for recurrent branches-to-PCs (red symbols) and main axons-to-DNCs (blues) from PND 15 rats. **(B)** shows linear correlations between sIPSC frequencies and amplitudes at CP_50_ for PCs (red symbols) and DNCs (blues) from PND 15 rats.

In the analysis of the functional correlations between presynaptic and postsynaptic partners, spike frequencies at PF_50_ in each axonal branch were read from samples in Figure 1E; and spikes per second at NS_50_ in each target cell were from samples in Figure 4B. Figure 7A shows the relationship between spike propagation fidelity on recurrent axons and spiking ability on Purkinje cells (red symbols), as well as that between propagation fidelity on main axons and spiking ability on deep nucleus cells (blues). The linearly proportional correlations in their functional states indicate that the abilities of processing spikes in axonal branches and their target cells are compatible.

In the analysis of the functional correlations between presynaptic GABA release and postsynaptic GABA receptor responses, sIPSC frequencies at CP_50_ were read from samples in Figure 6D; and sIPSC amplitudes at CP_50_ were read form Figure 6F). Figure 7B illustrates the linearly proportional correlations between GABA release from recurrent axons and GABAR responses in Purkinje cells (red symbols), as well as between GABA release from main axons and GABA response in deep nucleus cells (blues). These linearly proportional correlations indicate that the presynaptic GABA release and postsynaptic GABAR responses are compatible.

In addition to the functional differentiation between the pathway from recurrent axons to Purkinje cells and the pathway from main axons to deep nucleus cells, the presynaptic and postsynaptic partners in each pathway are compatible in their activity strength. Our studies firstly reveal that the axonal branches from each cerebellum Purkinje cell are functionally differentiated to be compatible with their postsynaptic partners. To validate this functional compatibility between presynaptic and postsynaptic partners, we have done these analyses in younger rats (postnatal day 8) and obtained the similar results (please see Supplementary Figures 1–4 in supporting data). The functional compatibility between presynaptic and postsynaptic partners in the different ages of animals indicates its natural presence and importance.

### Functional compatibility between axonal branches and target cells grants neural homeostasis

Physiological impacts for functional compatibility between presynaptic and postsynaptic partners may enable each neuron through its differentiated axonal branches to regulate their target cells properly, and their target cells to work efficiently for neuronal circuit homeostasis. We examined this hypothesis by a computational simulation. Purkinje cells and deep nucleus cells receive excitatory and inhibitory inputs. The strengths of GABAergic synapses in different presynaptic and postsynaptic partners were read from Figure 6. The abilities of encoding spikes in different target neurons were from Figure 4. The fidelities of spike propagation for main axons and recurrent axons were from Figure 1E.

**Figure 8:**
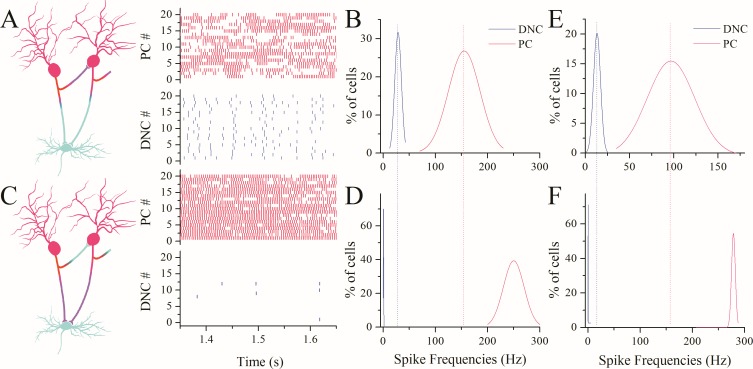
Functional compatibility between presynaptic and postsynaptic patterns makes the function of network neurons efficient **(A)** shows spike patterns at Purkinje cells (red vertical bars, top panel) and deep nucleus cells (blues, bottom) when recurrent branches vs. PCs and main axons vs. DNCs are functionally compatible (left panel, hot axons innervate active neurons, or vice versa). **(B)** shows the percentages of neurons vs. spike frequencies at PCs (red curve) and DNCs (blue). **(C)** shows spike patterns at Purkinje cells (reds, top) and deep nucleus cells (blues) when presynaptic functions are changed to recurrent axons-to-DNCs and main axon-to-PCs, i.e., functional incompatibility (left panel). **(D)** shows the percentages of neurons vs. spike frequencies at PCs (red curve) and DNCs (blue), where DNCs shifts toward functional silence and PCs toward overexcitation. **(E-F)** illustrates the percentages of neurons versus spike frequency (100 Hz) at PCs (reds) and DNC (blues) under the conditions of compatibility (E) and incompatibility (F) between presynaptic and postsynaptic partners.

Figure 8A–B illustrates the results from presynaptic and postsynaptic compatibility (left panel in 8A). The right-top panel in 8A shows spike patterns at Purkinje cells that are functionally compatible to recurrent axons. The right-bottom in 8A shows spiking patterns at deep nucleus cells that are compatible to main axons. Spike frequencies vs. number of Purkinje cells (red trace) or deep nucleus cells (blue) are plotted in Figure 8B. On the other hand, if the functions of recurrent branches and main axons exchange (left panel in Figure 8C), the spiking ability of Purkinje cells is upregulated to be synchrony (right-top panel) and the function of deep nucleus cells is almost silent (right-bottom). Statistical data are shown in Figure 8D. In addition to the firing frequency for Purkinje cells at 150 Hz, we also see the compatibility between presynaptic and postsynaptic partners when the firing rate of Purkinje cells is at 100 Hz (Figure 8E–F). The functional compatibility between the presynaptic and postsynaptic partners makes cerebellar networks coordinated; otherwise, the network neurons would be overexcited or functionally silent.

## DISCUSSION

The axon from a cerebellar Purkinje cell sprouts main axon and recurrent axons, which innervate deep nucleus cells and Purkinje cells, respectively. In this unit, the functional states of presynaptic axonal branches and of postsynaptic cells are differentiated. This differentiation makes a compatible relationship between presynaptic and postsynaptic partners (Figures 1, 2, 3, 4, 5, 6, 7). The functional compatibility makes active cells receiving more inhibitions, or vice versa, so that the neurons in each unit are activated appropriately (Figure 8). Our study reveals a new principle for cerebellar neural circuits to be functional in an optimal manner. Each neuron through its function-differentiated axon branches regulates divergent target neurons efficiently. The functional compatibility between the presynaptic and postsynaptic partners enables each microcircuit being homeostatic and entire circuits being coordinated for well-organized behaviors.

The cerebellar Purkinje cells produce high frequency spikes [38–41]. Their axonal branches innervate the divergent targets, e.g., deep nucleus cells and other Purkinje cells [42]. A connection divergence from a neuron to multiple cells expands the uses of its computational codes. In terms of their functional relationships, if inhibitory axonal branches inhibit inactive cells to be non-functional, the neural networks containing many silent neurons are inefficient. On the other hand, if inhibitory axonal branches innervate active cells, they may not inhibit these active cells, leading to neuronal synchrony in neural networks. The functional compatibility of presynaptic and postsynaptic partners makes postsynaptic cells to be regulated well and cerebellar neural network built optimally. In order to fulfill this goal, axonal branches from a Purkinje cell should be functionally differentiated to match their partner neurons. The functional compatibility in cerebellar network may play important roles in the cerebellum functions, such as behavioral coordination and flexibility [43].

The mechanisms underlying the functional differentiation and compatibility between presynaptic and postsynaptic partners are based on the various dynamics of voltage-gated sodium channels on these axonal branches and their target neurons (Figures 2 and 5) as well as of transmitter release and receptor responsiveness on these entities (Figure 6). It remains to be studied how the presynaptic and postsynaptic mechanisms in compatible manner are preset and regulated, whether the axonal branches and target cells form their synaptic connections by recognizing their functional compatibility or they reform coordinately after synapse formation, and whether the functional compatibility between presynaptic and postsynaptic partners is upheld during neural plasticity through anterograde and retrograde interaction.

It is noted that cerebellar Purkinje cells produce action potentials with a wide range of frequency from 50 to 250 Hz *in vivo* [38–41, 44]. Based on this information, the evoked spikes at the cerebellar Purkinje cells in our experiments were from 100 to 250 Hz in their frequencies, and the computational simulation matched this range of spike frequency (100–150 Hz in Figure 5). The indications in our study about the reliability of spike propagation and the compatibility of presynaptic versus postsynaptic partners are still suitable for establishing the concept under physiological conditions, in spite of closing to the high end of spike frequency.

In terms of neurons and synapses, many studies were focused on their plasticity and homeostasis [45–51]. The functional compatibility between presynaptic and postsynaptic cell partners should also be an important feature that makes cellular interaction coordinated and efficient, i.e., a principle for the optimal activities of neuronal networks. It is noteworthy that the different kinds of cells in tissue interact one another. The compatibility and coordination in their activity levels are critically important to maintain their appropriate interaction as well as not to waste the energy due to any difference in their activity levels.

It is noteworthy that several points should be stressed. In addition to voltage-gated sodium channels, voltage-dependent potassium channels are presumably to regulate spike refractory periods and neuronal excitability. Their differences can explain the functional differentiations of presynaptic axonal branches and their postsynaptic neurons. As the effect of potassium channels on refractory periods is done through sodium channels [28], the involvement of potassium channels in the functional differentiations, if it is present, may be fulfilled based on voltage-gated sodium channels. In addition to deep nuclear cells and Purkinje cells, the axonal branches of cerebellar Purkinje neurons terminate onto other types of postsynaptic neurons [17–19]. Whether the functional differentiation and compatibility are present on these units and partners should be done in our future studies.

The axonal branches that are functionally differentiated to match postsynaptic neurons constitute a fractional diverter without the need of intermediate components. This simplified design in the brain may be useful to build the compatibility among basic units in electronic circuits and social networks for their well-organized performance.

## MATERIALS AND METHODS

All experiments were performed in accordance with the relevant guidelines and regulations by the Administration Office of Laboratory Animals at Beijing China. All experimental protocols were approved by the Institutional Animal Care Unit Committee (IACUC) in the Administration Office of Laboratory Animals at Beijing China (B10831).

Brain slices and neurons: All experiments were approved by the Institutional Animal Care Unit Committee in Administration Office of Laboratory Animals Beijing China (B10831). Cerebellar sagittal slices (400 μm) were prepared from Wistar rats in postnatal days (PND) 8 or 14∼15 under the anesthesia by injecting chloral hydrate (300 mg/kg) for decapitation by a guillotine. Slices were cut by Vibratome in a modified and oxygenized (95% O_2_ and 5% CO_2_) artificial cerebrospinal fluid (mM: 124 NaCl, 3 KCl, 1.2 NaH_2_PO_4_, 26 NaHCO_3_, 0.5 CaCl_2_, 5 MgSO_4_, and 10 dextrose and 5 HEPES; pH 7.4) at 4°C, and were held in the normal oxygenated ACSF (mM: 126 NaCl, 2.5 KCl, 1.25 NaH_2_PO_4_, 26 NaHCO_3_, 2 CaCl_2_, 2 MgSO_4_ and 25 dextrose; pH 7.4) 35°C for 1∼2 hours before electrophysiological experiments. A slice was transferred to a submersion chamber (Warner RC-26G) and perfused by normal ACSF at 31°C for whole cell and loose patch recordings [23, 27, 29, 52–54].

Cerebellar Purkinje cells (PC) were identified based on their morphology and spiking properties. Purkinje cells in the slices (somata above 40 μm) for the whole-cell recordings were located at the border between molecular layer and granule cells. They were infused with a fluorophore Alexa-488 (5 μM in the recording pipettes) under a DIC-fluorescent microscope (Nikon, FN-E600) to show typical dendrites and to guide the tracing of axonal branches for loose-patch recordings. The Purkinje cells were also labeled by neurobiotin (Figure 1A). These Purkinje cells demonstrated fast spikes with no obvious adaptation in their amplitudes and frequencies [55–59].

Cerebellar deep nucleus was located at a convergent area of cerebellar lobes including excitatory and inhibitory neurons, and was innervated by the main axons of Purkinje cells [15, 19, 59]. Deep nucleus cells (DNC) appeared round soma and multiple processes under the DIC microscope (Nikon, FN-E600). The neurons in our analyses appeared fast spiking with no adaptation in the amplitudes and frequency, the typical properties for the interneurons [56, 59–64]. It is noteworthy that the reason to select inhibitory deep nucleus cells and Purkinje cells innervated by PC axonal branches in our study is to have the similar features of target cells, such that the differences in spiking propagation among PC axonal branches and in synaptic transmission at the pairs of PC-DNC and PC-PC are less likely affected by these PC-target cells.

Electrophysiological studies: *Sequential spikes in Purkinje cells propagate on their main axons and recurrent branches*. Each experiment in a Purkinje cell was conducted by whole-cell recording on its soma and two loose-patch recordings on the remote ends of its main and recurrent axons simultaneously (Figure 1A). The electrical signals were recorded by MultiClamp-700B amplifier (Axon Instrument Inc, CA USA) and inputted into pClamp-10 in 50 kHz sampling rate. Transient capacitance was compensated and output bandwidth was 3 kHz. Pipette solution for recording spikes included (mM) 150 K-gluconate, 5 NaCl, 0.4 EGTA, 4 Mg-ATP, 0.5 Tris- GTP, 4 Na-phosphocreatine and 10 HEPES (pH 7.4 adjusted by 2M KOH). The solution for loose-patch recording was ACSF (please see above). An osmolarity of pipette solutions made freshly was 295-305mOsmol. The pipette resistance was 8∼10MΩ [65, 66].

In studying spike propagation on the axonal branches of Purkinje cells, we injected depolarization pulses in various durations and intervals into their somata to induce spikes at 100, 150, 200 and 250 Hz. The spikes induced in these frequencies were based on the facts that cerebellar Purkinje cells fired high frequency spikes up to 500 Hz [38–41, 44]. The spikes propagated to axonal terminals were recorded by loose-patch at the remote ends of main axons and recurrent branches. Synchronous spikes at the soma and axon branches indicated signals from a Purkinje cell. The efficacy to propagate the spikes on the axons of Purkinje cells was assessed by a ratio of spikes recorded at axonal terminals to those induced on soma.

*The influences of the axonal branches of Purkinje cells on their target neurons* were evaluated by recording the events of GABAergic inhibitory synapses at adjacent Purkinje cells and deep nucleus cells. Spontaneous inhibitory postsynaptic currents (sIPSC) were recorded under a voltage-clamp [36, 67–70]. With the pipette solution composed of (mM) 135 K-gluconate, 20 KCl, 4 NaCl, 10 HEPES, 0.5 EGTA, 4 Mg-ATP, and 0.5 Tris–GTP, the Nernst's equation defined reversal potential at −43 mV for this Cl^−^ concentration, consistent with our recorded values. When cellular membrane potentials were held at −70 mV, sIPSCs were inward (down-fluctuation). 6-Cyano-7-nitroquinoxaline-2,3-(1*H*,4*H*)-dione (10 μM) and D-amino-5-phosphonovanolenic acid (40 μM) were added in the ACSF to block ionotropic glutamate receptors [36, 71, 72] and to record GABAergic IPSCs in isolation. At the end of each experiment, bicuculline (10 μM) was washed into the slices to test whether synaptic responses were mediated by GABA_A_R. sIPSC amplitudes represent the responsiveness of GABA_A_R, while sIPSC frequency reflects the innervation of GABAergic axons and the probability of GABA release. As the probability of GABA release from each axon is 100% (please see a paired-recording from an interneuron to another neuron in Supplementary Figure 2), sIPSC frequency may present the innervation of GABAergic axons. It is noteworthy that the recorded sIPSCs on Purkinje cells are likely generated from the axonal innervation from neighboring PC because the following reasons. The inhibitory synapses on cerebellar Purkinje cells dominantly come from neighboring PC than basket cells [73]. The recurrent axons of Purkinje cells terminate on the cell body of Purkinje cells, while the axons of basket cells terminate onto the dendrites of Purkinje cells, i.e., the propagations of PC-PC IPSCs to recording sites are less affected by passive membrane property. The frequency of spontaneous spikes is higher in cerebellar Purkinje cells than basket cells [38–41].

*The capability of the neurons to produce spikes* was assessed by measuring relationship between stimulus intensities and spikes (the input-output curve; [29, 31, 32, 74–76]. The capability of firing spikes was measured by counting spikes per second, while the stimulus intensities (i.e., depolarization pulses in one second) were increased in a step-by-step manner. As different neurons showed different excitability, the stimulus intensities were normalized for the data average. To each of the neurons, the spike threshold was detected by increasing stimulus intensities until seeing 50% chance to produce single spike by this stimulus. Absolute refractory periods (ARP) during the sequential spikes were measured by injecting paired-depolarization pulses (3 ms in duration and 1.2 times above threshold stimulus intensity, showed in Figure 2C) into the neurons after sequential spikes induced by a series of pulses (3 ms in duration and 200 Hz in frequency). By changing inter-pulse intervals, we defined ARP as the time from a complete spike to its subsequent spike at 50% probability [8, 35, 46, 52]. If the spikes during relative refractory period in the somata of Purkinje cells were able to be propagated to recurrent axon terminal but not main axon terminal, ARP would be longer in the main axons than recurrent axons.

The data were analyzed if the recorded neurons had resting membrane potentials more negative than −60 mV and action potentials at least 75 mV in amplitudes. The criteria for the acceptation of each experiment also included less than 5% changes in resting membrane potential, spike magnitude, input and seal resistance. The values of spike input-outputs and sIPSCs are presented as mean±SE. As experiments for different spike frequencies and control versus various treatments were conducted in the given cells, the statistical comparisons between groups are done by paired t-test.

Neurobiotin staining for cerebellar cells: Pipette solutions for whole-cell recordings included 0.2% neurobiotin, which were back-filled into the recording pipettes whose tips contained the standard solution. After electrophysiological study, the slices were rapidly placed into 4% paraformaldehyde in 0.1 M phosphate buffer solution (PBS) for fixation at 4°C about 48 hours. The slices were incubated in avidin and horseradish peroxidase (Vectastain ABC) for 3 hours, and then 1 % DAB–CoCl2 (Sigma) 1 min for staining neurobiotin-filled cells. This reaction was stopped by PBS [65]. Neurobiotin-stained cells were photographed under the DIC of the confocal microscope (Olympus FV-1000, Japan).

Computational simulation for neuronal activity in the cerebellum was done in NEURON (v7.1). Purkinje cells and deep nuclei cells in this study were innervated by excitatory and inhibitory synapses [10, 11, 13, 15, 17, 20]. In addition to the excitatory axons of parallel and mossy fibers, each Purkinje cell received inhibitory inputs from recurrent branches of 4∼5 adjacent Purkinje cells. The deep nuclei cells received inhibitory inputs from main axons of Purkinje cells. These neurons in the simulated network also received excitatory inputs from other areas in the central nervous system.

The factors inputted into the simulation were based on the properties of well-known network cells in the cerebellum and of inhibitory cells from our experiments. Each neuron in the simulated network was thought asan integrated-fire cell model in single compartment. Excitatory synaptic events were as steady inter-event intervals (5∼10ms). Functional properties for individual neurons to be inputted in a simulated network were based on our experimental data (Table 1).

**Table 1:** Physiological properties for cerebellar Purkinje cells and deep nucleus cells

Physiological properties	Purkinje cells		Deep nucleus cells	
	Biological	Model	Biological	Model
C_m_ (pF)	469.3±18.1	475	124.7±7.4	125
R_m_ (MΩ)	121.8±4.0	120	217.5±7.2	218
RMP (mV)	−60.5±0.7	−60.5	−49.2±1.0	−50
AP threshold potentials (mV)	−45.5±1.8	−45.5	−26.2±2.3	−26.2

To a role of ligand-gated ion channels in synaptic transmission, AMPAR and NMDAR mediated excitatory synapse activation, and GABA_A_R works for inhibitory synapses. Postsynaptic conductance was function as a sum of two exponentials (Equation 1) [77]. The values of synaptic conductance inputted into our simulated network were listed in Table 2 [78].

$$g_{syn}^{\left(t\right)}=\bar{g}*(\frac{\tau_{1}\tau_{2}}{\tau_{1}-\tau_{2}})*(e^{-\frac{\tau }{\tau_{1}}}-e^{-\frac{\tau }{\tau_{2}}})$$

**Table 2:** Functional dynamics of ligand-gated receptor channels in the synapses

Types of receptors	g_max_ (nS)	τ_1_(ms)	τ_2_(ms)	E_rev_ (mV)
**AMPA**	4	1	2	0
**NMDA**	19-27	0.67	80	0
**GABA_A_**	17-150	1	4	−70

In our simulation about the fidelity of spike propagation on axons in response to spike frequency, a relationship between spike frequency and propagation fidelity followed Equation 2, whose values were taken from Figure 1E. In this equation, f50 represents spike frequency caused 50% failure of action potential propagation and b is a constant. The parameters of Equation 2 were listed in Table 3.

$$p(f)=\{\begin{array}{cc} 1 & f<f_{50}<\frac{1}{b}\\ b\left(f_{50}+\frac{2}{b}-f\right) & f_{50}+\frac{1}{b}\leq f\leq f_{50}+\frac{2}{b}\\ & f<f_{50}<\frac{2}{b} \end{array}$$

**Table 3:** Parameters of action potential propagation fidelity

Types of cells	f_50_	B
Purkinje cell	255±2.18	0.0048±0.000045
Deep nucleus cell	220±6.16	0.0052±0.00014

## SUPPLEMENTARY MATERIALS FIGURES


