# Supplementary material for: Functional compatibility between Purkinje cell axon branches and their target neurons in the cerebellum
Source: Oncotarget. 2017 Aug 1;8(42):72424–37. doi: 10.18632/oncotarget.19770 (PMC5641142; doi:10.18632/oncotarget.19770)
Supplement: Supplementary file 1 [file oncotarget-08-72424-s001.pdf]

# Functional compatibility between Purkinje cell axon branches and their target neurons in the cerebellum

## SUPPLEMENTARY MATERIALS

## RESULTS

### Purkinje cell's axons and targets are functionally differentiated and compatible in young rats

The fidelity of spike propagation at Purkinje's axonal branches and the ability of spike production at Purkinje's target cells were analyzed in postnatal day 8 rats. Supplementary Figure 1 shows spike propagation fidelity on Purkinje's axonal branches and the spiking ability in their target cells. The plot for propagation fidelity versus spike frequency on main axons (blue symbols in Supplementary Figure 1) and on recurrent branches (red ones) shows that the fidelity of spike propagation is higher in recurrent branches than main axons ($p<0.01$; $n=8$ pairs). Supplementary Figure 2A illustrates spikes per second versus normalized stimuli for Purkinje cells (red symbols; $n=10$) and deep nucleus cells (blues; $p<0.01$, $n=10$). The abilities of processing spikes in the presynaptic and postsynaptic entities are differentiated in young rats.

Moreover, Supplementary Figure 3 illustrates the activities of GABAergic synapses on cerebellar Purkinje cells ($n=8$) and deep nucleus cells ($n=8$) in postnatal day 8 rats. sIPSC frequencies are higher in Purkinje cells (red symbols in Supplementary Figure 3A–B) than deep nucleus cells (blue), i.e., more GABA is released from Purkinje cell's recurrent branches than main axons. sIPSC amplitudes are higher in Purkinje cells (red symbols in Supplementary Figure 3C–D) than deep nucleus cells (blues), i.e., the higher responsiveness to GABA at Purkinje cells than deep nucleus cells.

To test whether the functional differentiations among presynaptic axon branches and among their target neurons establish a compatible relationship between presynaptic and postsynaptic partners in young rats, we plotted the relationships of spike propagation fidelity on Purkinje's axon branches versus spiking ability on their postsynaptic neurons, as well as the relationships between presynaptic GABA release and postsynaptic GABA receptor response. Based on data from PND 8 rats (Supplementary Figures 1–3), Supplementary Figure 4 shows that presynaptic and postsynaptic functions are linearly correlated. The functional compatibility between presynaptic and postsynaptic partners in the different ages of animals indicates its natural presence and importance.

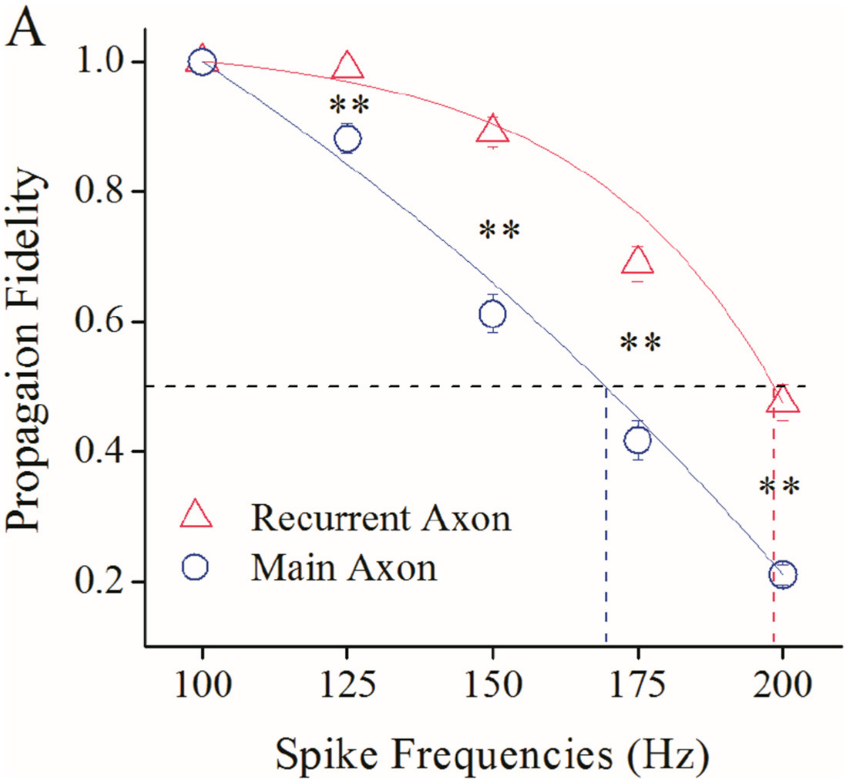


**Supplementary Figure 1: The differentiations in the fidelity of propagating spikes on distinct axon branches of cerebellar Purkinje cells are present in postnatal day 8 rats.** Somatic spikes were induced by depolarization pulses in 100, 125, 150, 175 and 200 Hz. The plot illustrates somatic spike frequency versus propagation fidelity (a ratio of axonal spikes to somatic ones) on the recurrent axons (red triangles) and the main axons (blues; two asterisks, $p<0.01$, $n=8$). Spike frequency at 50% of propagation fidelity ($FC_{50}$) is defined as spike-propagation efficiency.

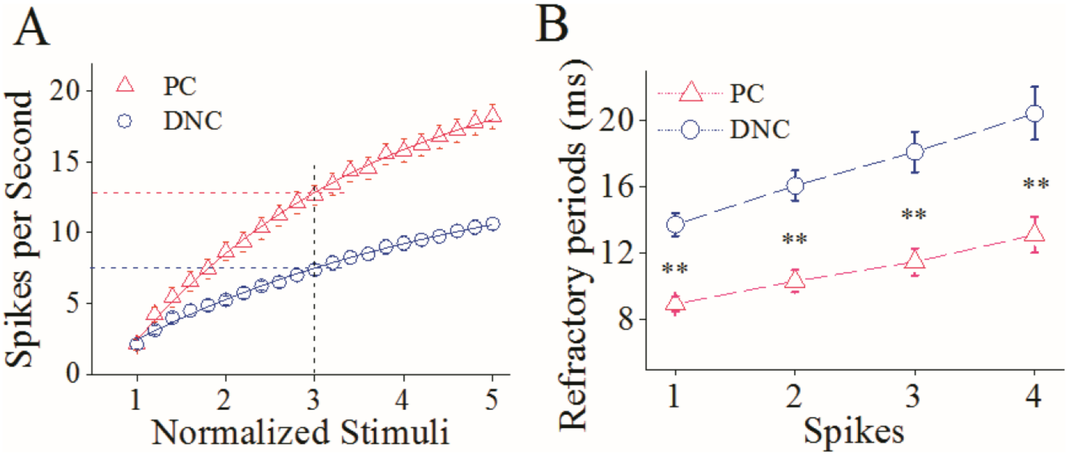


**Supplementary Figure 2: The differentiations in the ability of producing spikes are present on the different target cells innervated by the axonal branches of cerebellar Purkinje cells in postnatal day 8 rats.** The somatic spikes on cerebellar deep nucleus cells and Purkinje cells were induced by the different strengths of sequential depolarization pulses. **(A)** A plot illustrates spikes per second vs. normalized pulse intensities for Purkinje cells (red symbols, n=8) and deep nucleus cells (blues; $p<0.01$, n=8). **(B)** shows statistical data for RP values of spikes 1-4 at PCs (red symbols, n=8) and DNCs (blues, n=8; two asterisks, $p<0.01$).

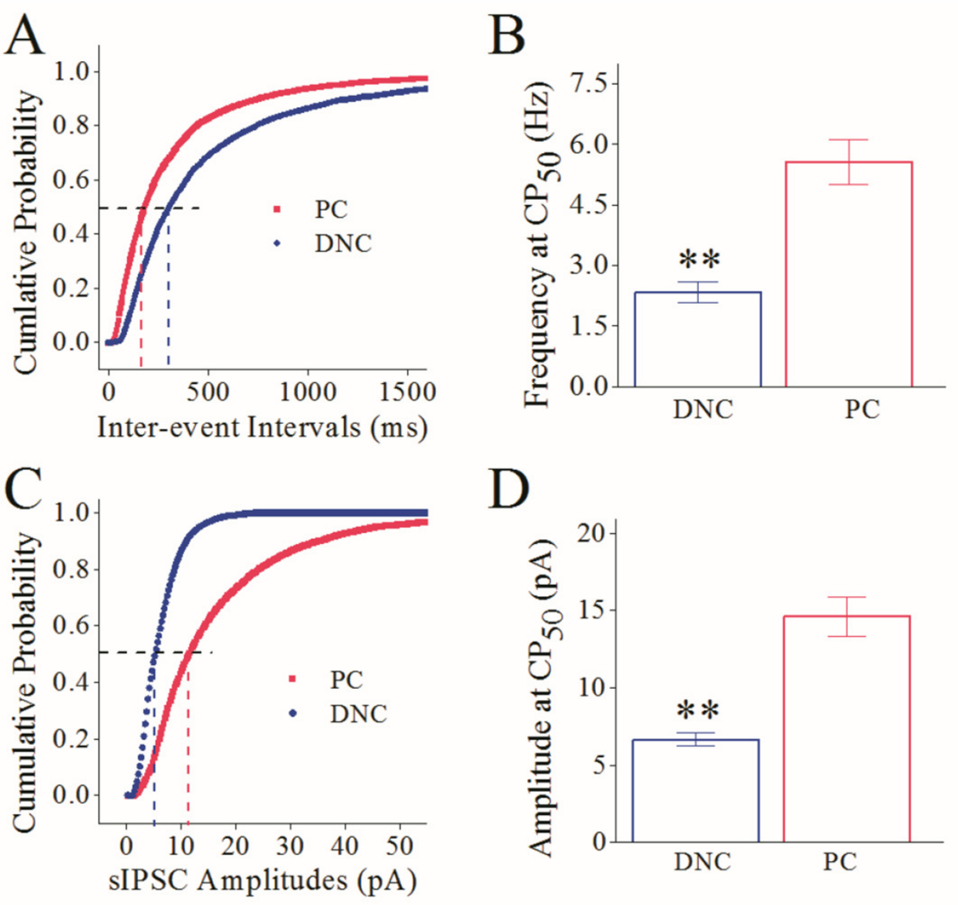


**Supplementary Figure 3: The activities of GABAergic synapses on cerebellar Purkinje cells (PC) and deep nucleus cells (DNC) in postnatal day 8 rats.** The activities of GABAergic synapses were evaluated by recording sIPSCs in the presence of CNQX/D-AP5. **(A)** shows cumulative probability versus inter-event intervals for DNCs (blue symbols, n=8) and PCs (reds, n=8). sIPSC frequencies (1/inter-event interval) at 50% cumulative probability ($CP_{50}$) denote GABA release. **(B)** shows sIPSC frequencies at $CP_{50}$ in recurrent axon (red bar) and main axon (blue; asterisks, p<0.01). **(C)** illustrates cumulative probability versus sIPSC amplitudes for DNCs (blue symbols, n=8) and PCs (reds, n=8). sIPSC amplitudes at 50% cumulative probability ($CP_{50}$) denote postsynaptic responsiveness. **(D)** illustrates sIPSC amplitudes at $CP_{50}$ on Purkinje cells (red bar) and deep nucleus cells (blue bar; asterisks, p<0.01).

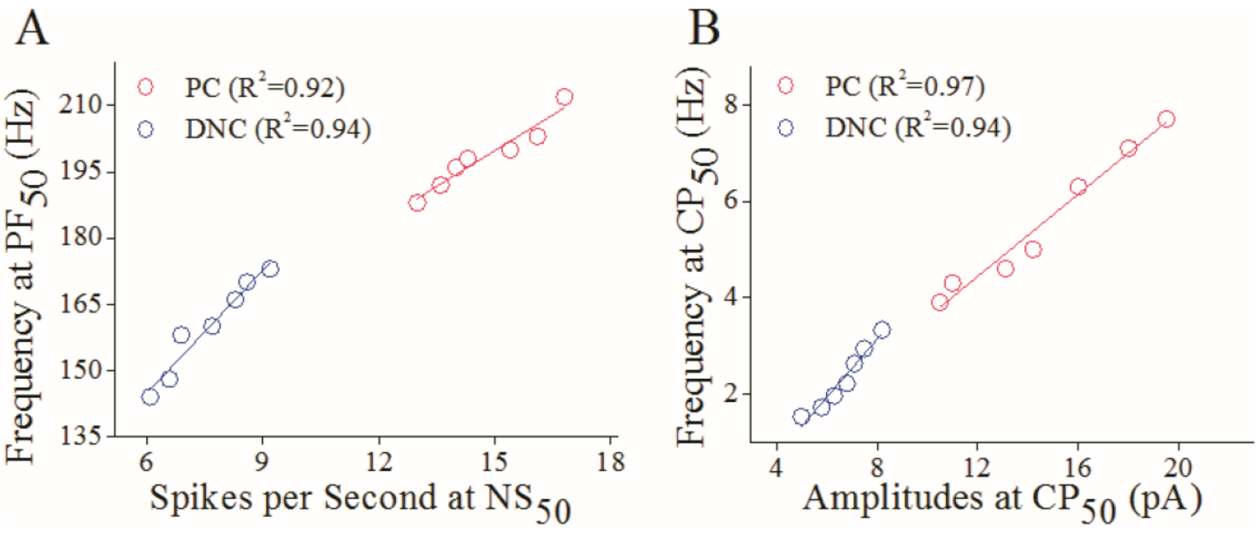


**Supplementary Figure 4 The correlations of functional status between presynaptic axonal branches of Purkinje cells (PC) and their target cells from PND 8 rats. (A)** shows linear correlations between presynaptic spike frequencies at $PF_{50}$ and postsynaptic spikes per second at $NS_{50}$ for recurrent branches-to-PCs (red symbols) and main axons-to-DNCs (blues) from PND 8 rats. **(B)** illustrates linear correlations between sIPSC frequencies at $CP_{50}$ and amplitudes at $CP_{50}$ for PCs (red symbols) and DNCs (blues) from PND 8 rats.
